# Instantaneous in vivo distal edge verification in intensity‐modulated proton therapy by means of PET imaging

**DOI:** 10.1002/mp.17850

**Published:** 2025-05-02

**Authors:** Brian Zapien‐Campos, Zahra Ahmadi Ganjeh, Giuliano Perotti‐Bernardini, Jeffrey Free, Stefan Both, Peter Dendooven

**Affiliations:** ^1^ Particle Therapy Research Center (PARTREC) Department of Radiation Oncology University Medical Center Groningen, University of Groningen Groningen The Netherlands; ^2^ Department of Radiation Oncology University Medical Center Groningen, University of Groningen Groningen The Netherlands

**Keywords:** intensity‐modulated proton therapy, Monte Carlo simulation, positron emission tomography, range verification

## Abstract

**Background:**

Intensity‐modulated proton therapy (IMPT) holds promise for improving outcomes in head‐and‐neck cancer (HNC) patients by enhancing organ‐at‐risk (OAR) sparing. A key challenge in IMPT is ensuring an accurate dose delivery at the distal edge of the tumor, where the steep dose gradients make treatment precision highly sensitive to uncertainties in both proton range and patient setup. Thus, IMPT conformality is increased by incorporating robust margins in the treatment optimization. However, an increment in the plan robustness could lead to an OAR overdosing. Therefore, an accurate distal edge verification during dose delivery is crucial to increase IMPT conformality by reducing optimization settings in treatment planning.

**Purpose:**

This work aims to evaluate, in a quasi‐clinical setting, a novel approach for accurate instantaneous proton beam distal edge verification in IMPT by means of spot‐by‐spot positron emission tomography (PET) imaging.

**Methods:**

An anthropomorphic head and neck phantom CIRS‐731 HN was irradiated at the head and neck region. The targets were defined as 4 cm diameter spheres. A 60‐ms delay was introduced between the proton beam spots in order to enable the spot‐by‐spot coincidence detection of the 511‐keV photons resulting from positron annihilation following the positron emission from very short‐lived positron‐emitting, mainly ^12^N (*T*_1/2_ **= 11.0 ms). Additionally, modified irradiations were carried out using solid water slabs of 2 and 5 mm thickness in the beam path to assess the precision of the approach for detecting range deviations. The positron activity range (PAR) was determined from the 50% distal fall‐off position of the 1D longitudinal positron activity profile derived from the 2D image reconstructions. Furthermore, Monte Carlo (MC) simulations were performed using an in‐house RayStation/GATE MC framework to predict the positron activity images and verify the PAR measurements.

**Results:**

PAR measurements achieved a precision between 1.5 and 3.6 mm (at 1.5σ clinical level) at the beam spot level within sub‐second time scales. Measured PAR shifts of 1.6–2.1  and 4.2‐–.7 mm were observed with the 2‐ and 5‐mm thickness range shifters, respectively, aligning with the corresponding proton dose range (PDR) shifts of 1.3–1.8 and 3.9–4.3 mm. The simulated PAR agrees with the measured PARs, showing an average range difference of ∼0.4 mm.

**Conclusion:**

This study demonstrated the feasibility of instantaneous distal edge verification using PET imaging by introducing beam spot delays during dose delivery. The findings represent a first step toward the clinical implementation of instantaneous in vivo distal edge verification. The approach contributes to the development of real‐time range verification aimed at improving IMPT treatments by mitigating range and setup uncertainties, thereby reducing dose to organs‐at‐risk and ultimately enhancing patient outcomes.

## 1 INTRODUCTION

The potential advantages of proton therapy over conventional x‐ray radiotherapy to treat deep‐seated tumors were first outlined in 1946.^1^ These advantages include the Bragg peak, which enables a high radiation dose deposition in targeted spots. Furthermore, the finite range of proton beams limits the dose to healthy tissues surrounding the tumor.^2^, ^3^ However, intensity‐modulated proton therapy (IMPT) conformality is very sensitive to range and setup uncertainties due to steep dose gradients at the target distal edge.^4^, ^5^ Currently, the best approach to increase the IMPT conformality is by the use of robustness settings in the treatment optimization.^6^ Nevertheless, an increase in plan robustness could lead to an OAR overdosing. Therefore, in order to maintain both normal tissue sparing and precision in the target coverage, it is necessary to accurately identify (preferably in real‐time) deviations between the dose distribution and proton range delivered during the patient irradiation and those planned through the treatment planning system (TPS). This describes what is known as dose and range verification.^7^, ^8^

Since the conception of proton therapy, range verification has been addressed by different approaches, one of them being positron emission imaging/tomography (PET). The possible applications of imaging the positron‐emitting tissue activation to visualize the end of the proton track (and determine the Bragg peak depth) were introduced in 1969 by means of helium ion beams.^9^ The principle of the technique is detecting in coincidence the 511‐keV positron annihilation photons created following the radioactive decay of positron emitters produced from the proton interaction with the tissues (mainly with carbon and oxygen nuclei) during the patient irradiation.

In‐room and in‐beam PET imaging for in vivo dose and range monitoring was investigated in the 1990s.^10^, ^11^ However, some issues were identified for online measurements (i.e. during the dose delivery) such as background radiation,^12^ detector saturation, low counting rate of 511‐ keV coincidences at the beginning of the irradiation, and the complex correlation between the positron activity and dose distribution.^13^ Due to the challenges presented by performing in‐beam PET image measurements, the technique has mainly focused on offline monitoring (i.e., after the irradiation has been delivered).^14^, ^15^ However, offline verification outside of the treatment room has its own drawbacks: potential changes in the patient position and the biological washout of the positron emitters during the time elapsed between their production during the irradiation and the image acquisition. These degrade the correspondence between the actual induced activity and the reconstructed positron activity image.^16^ In a quest for real‐time verification, several approaches based on prompt gamma rays have been studied (see^17^ for a recent review): prompt gamma imaging,^18^, ^19^ timing^20^ and spectroscopy,^21^ as well as acoustic‐based methods.^22^ A related method for in vivo range verification is range probing in which the measured integral depth‐dose curve of shoot‐through proton beams is compared with the calculation by the TPS.^23^, ^24^

Despite the above‐mentioned challenges for online PET imaging, some studies have made an effort to overcome them. Some of these studies have been performed both at centers for proton and carbon ion treatment, such as the GSI Helmholtz Centre for Heavy Ion Research, Germany,^25^ the National Cancer Center, Kashiwa, Japan,^26^ and the National Center of Oncological Hadrontherapy (CNAO), Pavia, Italy^27^ by means of the integration of PET imaging into the treatment environment. Because of the relatively poor quality of the PET data that was acquired while the beam was present, such data has not been used for irradiation verification. This means that at the cyclotron‐based facility in Kashiwa, only PET data recorded after irradiation were used, and in the synchrotron‐based facilities GSI and CNAO, only PET data recorded during the 2–4 s pause in‐between 1–2 s beam “spills” were used. At synchrotron‐based facilities, PET imaging on‐the‐fly during irradiation has been demonstrated, with an average delay of 6 s between beam delivery and image availability.^27^ Cumulative PET imaging was demonstrated: at each image update (in time steps of 10 s in^27^), all data acquired since the start of the irradiation is used. The main contributing positron emitters, ^15^O and ^11^C, have relatively long half‐lives of 2 and 20 min. Thus, as the irradiation progresses, the average time since the creation of the positron‐emitting nuclides whose decay is seen during a time step increases. Therefore, biological washout effects play an increasing role as the irradiation progresses.

In recent years, we have investigated the usefulness of imaging the very short‐lived positron emitter ^12^N (*T*_1/2_ **= 11.0 ms) for verification of proton therapy. By subtracting the longer‐lived contribution from the acquired PET data, pure ^12^N images can be obtained,^28^ providing instantaneous feedback on the irradiation at the sub‐second time scale (“instantaneous” refers to the very short time between the creation of ^12^N by the proton beam and the detection of its decay providing feedback information). Such instantaneous feedback is not possible for ^15^O and ^11^C: even though their production cross‐section maxima are about 20 times larger than that of ^12^N, only 0.6% of ^15^O and 0.06% of ^11^C decay within 1 s of their creation. At the beginning of irradiation, the contribution of ^12^N to the PET activity is the main one.^29^ As irradiation progresses, the contribution to the PET data from longer‐lived isotopes increases and, in general, the number of protons in a pencil beam spot decreases (assuming that, as in clinical practice, the energy layers are delivered in order of decreasing proton energy). Therefore, the quality of ^12^N information deteriorates as irradiation progresses. Because of this, we only focus on ^12^N imaging for verification during the first few seconds of irradiation. This typically corresponds to the first few highest energy layers delivering the distal edge dose. Our aim is thus instantaneous distal edge verification.

Using fixed pencil beams irradiating homogeneous phantoms, we have up to now imaged the ^12^N positron activity at the level of individual beam spots, enabling to determine the proton range with a 1σ uncertainty of 5 to 1 mm (using 10^8^ to 10^10^ protons per spot).^30^

In clinical practice, verification will be based on comparing measured PET images with those predicted based on the treatment plan. We have recently implemented such prediction tools by measuring the ^12^N production cross‐section versus proton energy^31^ and incorporating it in a RayStation/GATE MC simulation framework for range verification.^32^

The planning and execution of proton therapy incorporate many features to ensure sufficient tumor control with minimal side effects due to the irradiation of healthy tissue/organs‐at‐risk. These features include the safety margins used in robust optimization algorithms in IMPT treatment planning, which are 3%–5% for range uncertainty and 3–5 mm for setup errors.^33^, ^34^ These margins ensure a safe treatment, no matter what the deviation of the Bragg peak position or the patient position within the margins established by means of quality control procedures. Feedback on the proton range within the first couple of seconds of irradiation, thus verifying the distal edge of the dose distribution and enabling interruption of the irradiation in case of a too‐large deviation from the treatment plan, may enable the use of smaller margins in robust treatment planning. It may be a way to reduce the safety margins, resulting in better dose distributions, without compromising treatment safety. Reducing the robust planning margins has been shown to reduce the normal tissue complication probability (NTCP) without modifying the tumor dose coverage.^35^, ^36^

In the present study, we make an essential step in the translation to the clinic of ^12^N imaging for instantaneous distal edge verification during proton therapy, in terms of the target, the beam delivery and the comparison with the treatment‐plan based prediction. We determine the accuracy with which the range of individual beam spots of the two most distal energy layers can be verified via PET imaging in the irradiation of an anthropomorphic phantom irradiated according to a quasi‐clinical treatment plan and compare the measurements with the predictions from a treatment‐plan based MC simulation framework.

## 2 METHODS

### 2.1 Treatment planning workflow and irradiations

The phantom used was a CIRS Proton Therapy Dosimetry Head Model 731‐HN (CIRS, Norfolk, USA), an anthropomorphic head phantom designed for commissioning and dosimetric verification in IMPT systems.^37^

#### 2.1.1 Phantom CT acquisition

**FIGURE 1:**
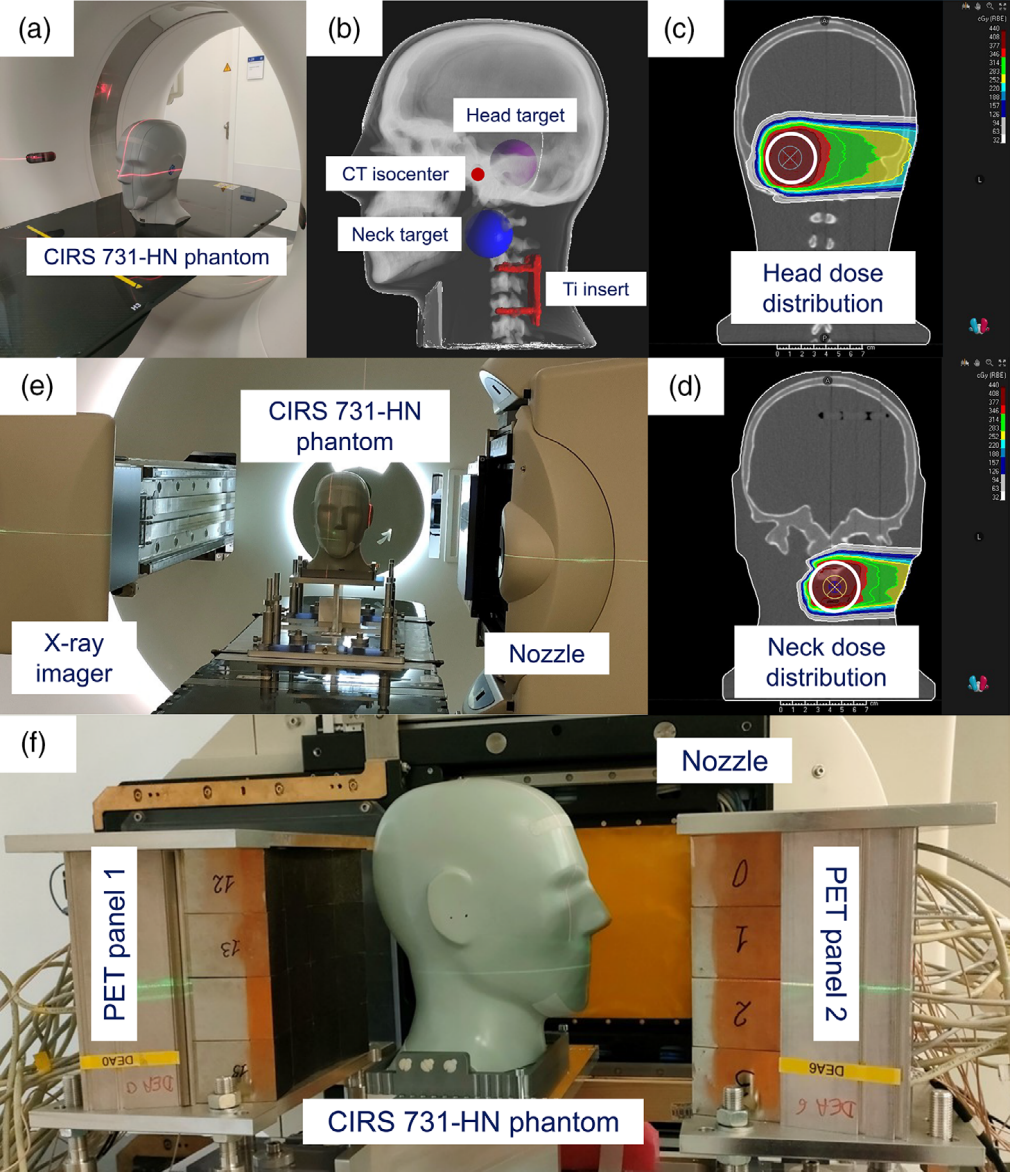
Illustration of the experimental setup used in this study. (a) CIRS 731‐HN phantom positioned within a Siemens SOMATOM Definition AS Open CT Scanner for planning CT acquisition. (b) Sagittal phantom view indicating head (pink sphere) and neck (blue sphere) target location, planning CT isocenter (red point), and titanium insert (red structure). (c) Head plan dose distribution in coronal view. (d) Neck plan dose distribution in coronal view. In (c) and (d), the white circle represents the central cross‐section of the targets. (e) Setup of the phantom in the treatment room with the on‐board x‐ray imager for positioning verification. (f) Experimental setup: Gantry at 90°, upright CIRS 731‐HN phantom, PET panels positioned perpendicular to the beam direction with a distance of 40 cm in between them.

The phantom planning CT was acquired using a Siemens SOMATOM Definition AS Open Scanner (Siemens Healthineers, Germany). Unlike the general IMPT workflow, the planning CT was acquired in upright position in order to match the current experimental setup. The CT was acquired using the standard protocol for HNC patients: a tube voltage of 120 kV, FoV of 500 mm, pixel size of 0.98×0.98 mm^2^, and 0.6 mm slice thickness (Figure 1a).

#### 2.1.2 Target definition

Clinical target volumes (CTVs) were defined in a research version of RayStation TPS v11‐B(R) (RaySearch Laboratories, Stockholm, Sweden). The targets were defined at two positions, head and neck, as a 4 cm diameter sphere (Figure 1b). The head CTV isocenter was located at (4, 8, 48 mm) and the neck CTV isocenter was located at (−29, 38, 10.8 mm), referring to the imaging convention (Right‐Left, Inferior‐Superior, Posterior‐Anterior) and relative to the planning CT isocenter (i.e., the CT scanner isocenter).

#### 2.1.3 Treatment plan

From this point forward, all doses will be expressed in terms of relative biological effectiveness (RBE) dose, with an RBE = 1.1. Plans were generated in the TPS using the following objective functions: a uniform dose of 400 cGy, maximum dose of 408 cGy, and minimum dose of 392 cGy, and for the neck plan, a restrictive function of 10 cGy at 10% of the volume of the titanium insert in order to avoid overdosage and the production of positron emitters in the metal structure. We delivered a single‐field dose of 4 Gy, but in a real clinical scenario, the typical fraction dose is 2 Gy distributed across 3–6 fields. This means that the most intense field delivers at most 1 Gy to the CTV. Consequently, the delivered dose was approximately four times higher than what would be expected in a clinical setting. The reason for this choice is that, as we have shown in previous work,^30^ the uncertainty of the range verification method is essentially determined by the number of PET counts (the uncertainty scales with the inverse square root of the number of counts). The dose of 4 Gy compensates for the fact that our setup is non‐optimal with respect to the number of counts in two ways: (1) the size of the PET panels, as well as their angular coverage, can be increased; (2) ^12^N decays are not detected during the beam spots, and as these have a duration on the order of the ^12^N half‐life, this is a sizeable loss. We estimate the loss in the number of counts due to these two points to be about a factor of four. The treatment plan consisted of 987 beam spots distributed over 17 energy layers ranging from 102.7 to 146.5 MeV for the head plan and 737 beam spots distributed over 23 energy layers ranging from 70.0 to 109.3 MeV for the neck plan. The dose distribution of both head and neck treatment plans is shown in Figure 1c,d. The spot‐by‐spot 3D proton dose distributions were exported from the RayStation TPS simulation to calculate 1D longitudinal projections. The proton dose range (PDR), defined as the position of the 80% dose level on the distal projection distal, was determined by linear interpolation. These values worked as the ground truth for the discussion of the results.

#### 2.1.4 Beam spot delay implementation

In the default clinical settings for pencil beam spot scanning, an unavoidable delay of a few milliseconds can be found between spots. However, this delay is too short for an individual per‐spot ^12^N data acquisition because of the photomultiplier tube (PMT) recovery time of a few milliseconds and the ^12^N half‐life being a few times longer than the delay.^30^ To enable per‐spot ^12^N data acquisition without mixing data from previous spots, a pause of 60 ms was added by adjusting the default slew settings of the beam delivery system.

#### 2.1.5 Phantom positioning

The head phantom was positioned on the treatment couch, and the position was verified by an x‐ray planar image using the on‐board cone beam CT (CBCT) equipment. A six‐degree‐of‐freedom (6‐DoF; lateral, longitudinal, vertical, yaw, pitch and roll) geometry accuracy evaluation was performed in order to minimize the errors between the x‐ray planar image and the digitally reconstructed radiography (DRR) generated from the planning CT. The positioning verification suggested small corrections (translational shifts of less than 1 mm and rotational shifts of less than 0.2°), which were applied (Figure 1e).

### 2.2 PET scanner

The scanner used in this work is a dual‐head PET scanner based on the Siemens Biograph mCT^38^ clinical scanner. Details have been reported in our previous studies on ^12^N PET imaging.^30^, ^31^, ^32^ The panels were attached to the treatment couch by means of custom‐made support to have a center‐to‐center panel distance of 40 cm. The isocenter of the CTV was aligned with the PET scanner in the lateral and vertical directions (Figure 1f).

### 2.3 PET data acquisition and image reconstruction

The PET data consisted of a list‐mode acquisition of coincidence events, with a time stamp resolution of 1 ms. An energy window of 430–650 keV and a coincidence time window of 5 ns were set. In our previous studies at the Particle Therapy Research Center (PARTREC) in Groningen, both beam pulsing and pulsing of the PET scanner PMTs were controlled by a user‐generated signal. At the Groningen Proton Therapy Center where the present work was performed, the duration of the beam spots varies as determined by the standard clinical beam delivery and no signal is available to control the PMTs in synchronization with the beam spot delivery. We, therefore, employed a continuous acquisition but did not use the data acquired during the beam spots in the analysis. For this purpose, the beam spot periods can be easily identified from the time histogram of the list mode data (see Section 3.1).

To determine the background activity, there was no beam during the first 60 s of data acquisition. Then, the treatment plan was delivered, lasting around 80 s. After treatment plan delivery, post‐irradiation PET data were acquired up to a total data acquisition time of 660 s.

The image reconstruction consisted of the 2D histogram of the line‐of‐response (LOR) intersections with a chosen reconstruction plane.^28^ The reconstruction plane was located in the middle, between the two panels. The images were segmented into pixels of 8×8 mm^2^ in order to reduce the statistical uncertainty of the individual pixel values. No attenuation or sensitivity corrections were applied on both measured and simulated images in order to avoid the error propagation on the profiles due to the corrections and thus perform the comparison as reliably as possible.

### 2.4 Monte Carlo simulations

**FIGURE 2:**
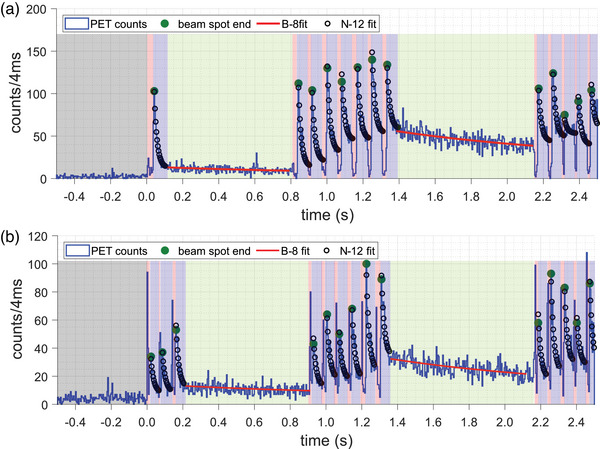
Time histogram of the PET counts during the first 2.5 s (and 0.5 s prior) of the 4‐Gy head (a) and 4‐Gy neck (b) irradiation. The following structures are observed: individual beam spots (red shaded areas), beam spot delay (beam‐off period) of about 60 ms (blue shaded areas), and the energy layer switch of about 700–800 ms (green shaded areas).

The Monte Carlo simulations were performed using a dedicated RayStation and Geant4 Application for Tomographic Emission (GATE)^39^ MC simulation framework.^32^ The workflow to obtain the simulated PET image per spot was the following:

1. The treatment plan was imported in the RayStation TPS v11B‐IonPG (version 12.0.130) (RaySearch Laboratories AB, Sweden) in order to calculate the induced positron activity, a feature that was developed in a collaboration between RaySearch Laboratories and the Ludwig Maximilians Universität München, initially for the analytical^40^ and later for the MC dose engine,^41^ including the possibility to import external cross sections and, in the MC version, to do scoring per spot. The reaction channels included in this simulation were ^12^C(p, pn)^11^C, ^16^O(p, 3p3n)^11^C, ^16^O(p, pn)^15^O, and ^12^C(p, n)^12^N, the latter being added for this study.^31^ The number of protons simulated was 8×10^5^ for the head irradiation and 9×10^5^ for the neck irradiation distributed over all the spots of the first two energy layers according to their relative weight (Tables S1 andS2).
2. The positron yield distributions and the planning CT were imported into our in‐house GATE code to simulate the transport of positrons and 511‐keV annihilation gamma rays through the phantom and PET system in order to get the list‐mode files of the coincidence events per spot and radionuclide. Three scenarios, the nominal and the two shifted, were simulated for each plan. Each scenario consisted of the simulation of the three isotopes mentioned above for the first eight and nine spots of the head and neck plan, respectively. The number of decays simulated per scenario was 4.0×10^8^ and 4.5×10^8^ for the head and neck plan, respectively. These decays were distributed among the isotopes produced by each spot according to the production calculated by the simulation performed in RayStation. These simulations were performed on a personal computer equipped with an 8‐core CPU. The total runtime was 108 h.
3. Image reconstructions were performed using the same method as described for the experimental measurements (see Section 2.2).
4. Due to the MC engine just simulating the isotope production, the total PET image does not correspond to the sum of the individual production images for the different radionuclides. Scaling of these images by decay factors which depend on the radionuclide half‐life and the time structure of the irradiation is needed. The time structure was obtained from the treatment‐specific irradiation log files, which have a time resolution of 200 µs. Furthermore, the time structure was confirmed by the PET activity histograms (Figure 2). Equations (1) and (2) show the weighting factors taken into account to get the total PET image for each beam spot.
  4.1 ^12^N image contribution to the i‐th spot: $I_{12N,i}$
(1) $$\begin{array}{cc} I_{12N,i} & =N_{p,i}\times \left(\frac{1-\exp(-\lambda_{12N}t_{0,i})}{t_{0,i}}\right)\\ & \;\times \left(\frac{1-\exp\left(-\lambda_{12N}t_{D}\right)\;}{\lambda_{12N}}\right)\times P_{12N,i} \end{array}$$

where $N_{p,i}$ is the number of protons of the i‐th spot obtained from the treatment plan, $\lambda_{12N}=63.01\;s^{-1}$ is the decay constant of ^12^N, $t_{0,i}$ is the i‐th spot duration obtained from the irradiation log files, $t_{D}=60\;\mathrm{ms}$ is the duration of data acquisition, and $P_{12N,i}$ is the ^12^N production image obtained from the GATE simulation. Due to the very short half‐life relative to the time between spots, the ^12^N activity induced during one spot does not contribute to the counts of the subsequent spot.

4.2 ^11^C and ^15^O image contribution to the i‐th spot:$I_{X,i}$
(2) $$\begin{array}{cc} I_{X,i} & =\sum_{j=0}^{i}N_{p,j}\times \lambda_{X}\times t_{D}\\ & \;\times \exp\left(-\lambda_{X}\left(t_{e,i}-t_{e,j}\right)\right)\times P_{X,j} \end{array}$$
  where $N_{p,j}$ is the number of protons of the j‐th spot obtained from the treatment plan, $\lambda_{X}$ is the decay constant ($5.67\times 10^{-4}\;s^{-1}$ for ^11^C and $5.67\times 10^{-3}\;s^{-1}$ for ^15^O), $t_{e,i}-t_{e,j}$ is the time between the end of the delivery of the i‐th and j‐th spot obtained from the irradiation log files, and $P_{X,j}$ is the ^11^C or ^15^O production image obtained from the GATE simulation. Unlike the previous case, the ^11^C and ^15^O activity induced during one spot does contribute to the counts of the subsequent spots, which is why the summation operator appears in Equation (2). Radiation washout was not included in this study because this was performed on a phantom. On the other hand in in vivo studies, the washout would also be negligible due to the short time scales of the acquired images.
4.3 Finally, the total spot image is given by the sum of these three main contributions: $I_{\mathrm{Tot},i}=M\left(I_{12N,i}+I_{11C,i}+I_{15O,i}\right)$, where M is a constant introduced to match the experimental total number of counts because of the unknown normalization of the positron yield distributions in the RayStation TPS.

### 2.5 Shifted irradiation

Next to the nominal irradiations, two range‐shifted irradiations were performed. The proton range was shifted by a 2 and a 5 mm thick RW3 solid water slab (SP34, IBA Dosimetry, Schwarzenbruck, Germany) between the nozzle and the head phantom. The composition of the range shifter is 98% polystyrene (C_8_H_8_)_n_ and 2% TiO_2_ and a density of $1.045\;g/cm^{3}$. Simulations were performed including the solid water slabs in the RayStation TPS.

### 2.6 Data analysis

The data analysis of both simulations and measurements was performed on the first two energy layers of the irradiations. These layers comprise the beam spots with the largest number of protons and define the distal edge of the dose distribution, which is the primary aspect being verified in this study.

**FIGURE 3:**
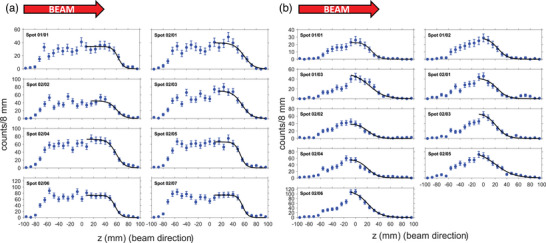
1D longitudinal positron activity profiles of individual beam spots: (a) First eight spots (one spot from the 146.5‐MeV energy layer and seven from the 143.5‐MeV energy layer) delivered during the head irradiation. (b) First nine spots (three from the 109.3‐MeV energy layer and six from the 107.0‐MeV energy layer) delivered during the neck irradiation. The blue dots show the PET counts detected and the black line the sigmoid function (Equation 3) fitted to the distal fall‐off region. The spot ID is represented by the code: XX/YY, where XX is the energy layer number and YY is the spot number, both in delivery order. The z‐axis indicates the position relative to the CT phantom isocenter (see Figure 1). The error bars indicate the 1σ statistical uncertainty calculated as the square root of the number of counts.

1D longitudinal positron activity profiles were obtained by projecting both measured and simulated reconstructed images along the lateral direction. Then, the positron activity range (PAR) was determined as the location at the 50% distal fall‐off position of a sigmoid function that was fitted by means of weighted least‐square fitting (MATLAB R2022b) to the 1D profiles. The sigmoid function is given by:

(3) $$A\left(z\right)=\frac{A_{0}}{1+\exp\left(\frac{z-\mathrm{PAR}}{r}\right)\;}$$

where A(z) is the distal activity profile in counts/pixel and z the beam direction coordinate (z=0 mm indicates the planning CT isocenter). The free parameters of the fit were: A_0_, the amplitude (in counts per pixel) of the sigmoid function; PAR (in mm), the z‐location where 50% of the sigmoid amplitude is reached, and r, indicating the rate (in mm) at which the edge of the sigmoid function falls off. We take the 1σ uncertainty on the PAR fit parameter given by the fitting algorithm as the uncertainty on the PAR. The end point of the fitting range was selected at z = +96 mm, a location beyond the Bragg peak, where the positron activity profile is essentially zero (see Figure 3). To determine the appropriate starting point of the fitting range, we conducted some tests to assess how the fit parameters vary based on this starting point. For the head irradiation, the parameter variation when changing the starting point from z = +8 to +48 mm (fully covering the target location) was smaller than the uncertainty of the fit parameter, while for the neck irradiation the parameter variation was smaller than the uncertainty of the fit parameter when changing the starting point from z = ‐8 to +8 mm (the central region of the target location). To ensure the parameter stability and to maximize the number of data points used for the fitting, we selected as the starting point of the fitting range z = +8 mm for the head irradiation and z = ‐8 mm for the neck irradiation. Therefore, the results do not significantly depend on the exact choice of the fitting range. Goodness‐of‐fit was evaluated by a two‐tailed chi‐square (χ^2^) test, which indicated no significant difference between the observed and expected values at a significance level of *p *< 0.05.

## 3 RESULTS

### 3.1 Spot‐level data acquisition

Figure 2a shows the time histogram of the PET counts from 0.5 s prior to and during the first 2.5 s of a 4‐Gy head irradiation on the phantom, showing the first two energy layers of 146.5 and 143.5 MeV and a part of the third energy layer (140.5 MeV). Figure 2b shows the histogram for a 4‐Gy neck irradiation, showing the first two energy layers of 109.3 and 107.0 MeV and a part of the third energy layer (104.8 MeV). The individual beam spots (red‐shaded areas) are identifiable because the count rate drops suddenly due to the saturation of the PMT‐based detectors.

During the beam spot delays (blue‐shaded areas), a decay with a short half‐life was observed, which, by means of a half‐life analysis, indicates the presence of ^12^N counts (black circles). Once all spots of the same energy layer have been delivered, the layer switches to lower energy, resulting in a beam‐off period lasting around 700 ms (green‐shaded areas) where counts of ^8^B (*T*_1/2_ **= 770 ms) and a background component stemming from relatively long half‐lived positron‐emitters (^11^C and ^15^O) were identified through a half‐life analysis.

For the beam spots of the first two energy layers, the ^12^N activity is substantial compared with the other activity, which is essentially from the long‐lived positron‐emitting ^11^C (*T*_1/2_ **= 20.4 min), ^15^O (*T*_1/2_ **= 2.04 min), ^10^C (*T*_1/2_ **= 19.3 s), and ^8^B (*T*_1/2_ **= 770 ms). For the first energy layer, the ^12^N counts were the majority of coincidence events detected for both the head and the neck irradiation. The total number of counts (^12^N and the other positron emitters) detected per spot during the first 60 ms of the PET acquisition time between spots was between 570–1237 and 238–559 for the 4‐Gy head and neck irradiation, respectively. Details on the relative contribution of ^12^N, ^11^C, and ^15^O activities are discussed in Section 3.2.

Figure 3a shows the activity profiles of the first eight delivered spots for the 4‐Gy head irradiation, one from the 146.5‐MeV energy layer and seven from the 143.5‐MeV energy layer, with 2.80–5.28×10^8^ protons per spot. Figure 3b shows the activity profiles of the first nine delivered spots for the 4‐Gy neck irradiation, three from the 109.3‐MeV energy layer and six from the 107.0‐MeV energy layer, with 1.09‐3.89×10^8^ protons per spot.

### 3.2 Monte carlo simulations

**FIGURE 4:**
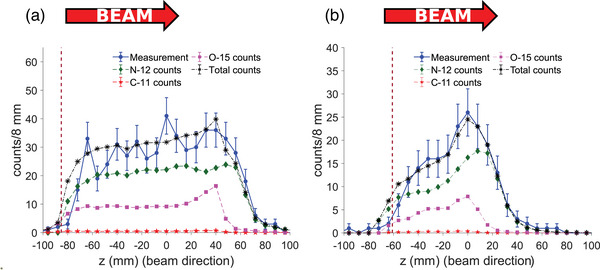
1D longitudinal positron activity profile for the first (and most distal) spot: (a) For head irradiation, the first beam spot consisted of 5.28×10^8 ^protons of 146.5 MeV. (b) For neck irradiation, the first beam spot consisted of 2.27×10^8^ protons of 109.3 MeV. Measured profile (blue dots), simulated profiles: total counts (black asterisks), ^12^N counts (green diamonds), ^15^O counts (pink squares), and ^11^C counts (red stars). The brown dotted vertical line represents the position of the beam spot entry into the phantom. This position is −85.2 and −60.9 mm for the head and neck irradiations, respectively. The error bars indicate the 1σ statistical uncertainty calculated as the square root of the number of counts.

Table S1 (for the 4‐Gy head irradiation) and Table S2 (for the 4‐Gy neck irradiation) present, for each spot, the relative contribution of each positron‐emitting isotope to the detected PET counts, which were derived from MC simulations. Figure 4 compares the measured (blue dots) and the simulated (black asterisks) 1D longitudinal activity profiles of the first spot (and most distal) for the head (left) and the neck (right) irradiation. Because of the unknown normalization of the positron activity calculated by the RayStation MC engine, the simulated PET image was normalized to match its total counts with that of the measured PET image. Although the overall ^15^O contribution is non‐negligible, the distal edge of the positron emission activity profile is fully determined by the ^12^N activity. Figure S7 shows the 2D image reconstruction for the first spot delivered in the head and neck nominal irradiation from which the profiles in Figure 4 were calculated.

### 3.3 Range and shift measurements

**FIGURE 5:**
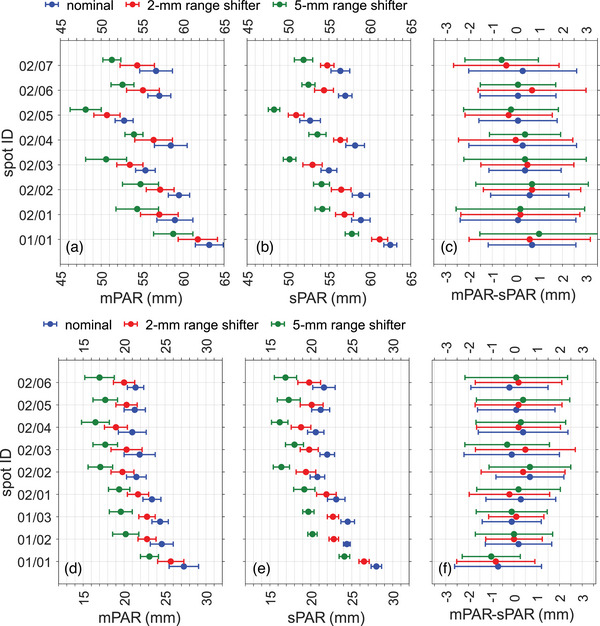
(a) and (d) mPAR of the 4‐Gy head and neck irradiation for the nominal and shifted irradiations, (b) and (e) sPAR of the 4‐Gy head and neck irradiation for the nominal and shifted simulations, (c) and (f) difference between mPAR and sPAR, showing the validation of the MC simulations. The difference is substantially smaller than the combined range uncertainty. The horizontal error bars indicate the 1σ statistical uncertainty.

Figure 5a,d show the measured positron activity range (mPAR), and Figure 5b,e show the simulated positron activity range (sPAR) from the nominal and shifted irradiations. These data are also presented in Tables S3–S8. The measured PAR shift is compatible with the simulated PAR and PDR shifts within their respective uncertainties, as shown in Table S9. For individual spots in the first two energy layers, the 1σ uncertainties on the mPAR values were 1.1–2.4 mm for the 4‐Gy head irradiations and 1.0–1.9 mm for the 4‐Gy neck irradiations. Additionally, Figure 5c,f show the difference between the measured and simulated PAR. The average absolute difference is 0.40 mm and the average uncertainty (1σ) 2.1 mm for the head irradiation, and the average absolute difference is 0.3 mm and the average uncertainty (1σ) 1.5 mm for the neck irradiation.

**FIGURE 6:**
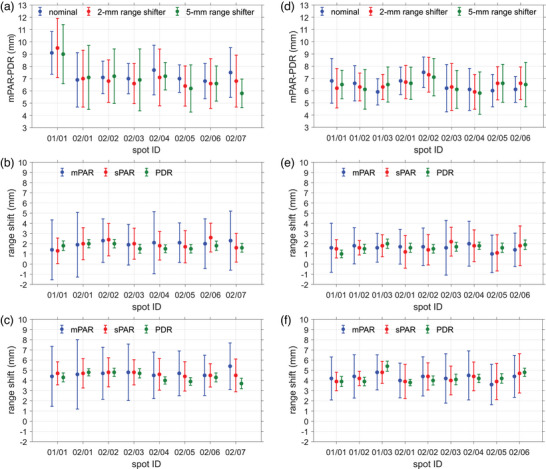
Difference between the mPARs and the PDRs for the nominal and shifted setups for both cases, (a) head and (d) neck irradiation. mPAR, sPAR, and PDR shifts for (b) 2‐mm range shifter in head irradiation, e) 2‐mm range shifter in neck irradiation, (c) 5‐mm range shifter in head irradiation and (f) 5‐mm range shifter in neck irradiation. These show the first eight spots of the head irradiation and nine spots of the neck irradiation. The error bars indicate the 1‐σ combined uncertainty.

Figure 6a,d show an expected systematic difference between the mPARs and the PDRs. The difference between mPAR and PDR is between 6.4 and 7.7 mm for the head irradiation, except for the first spot, whose difference is 9.0–9.5 mm, whereas for the neck irradiation, the difference is between 5.9 and 7.5 mm. There is no physical reason to justify why the first spot of the head irradiation presented a ∼2 mm larger difference. Therefore, a systematic error is very likely to have occurred in the measurement or data analysis of this spot. The differences between mPAR and PDR show an average value (without the measurement of the first spot) of 6.9 mm and a standard deviation of 0.5 mm for the head irradiation, and an average value of 6.4 mm and a standard deviation of 0.4 mm for the neck irradiation. This implies that the inter‐spot variations are about two to five times smaller than the PAR uncertainty of individual spots, suggesting that the actual uncertainty could be lower than that calculated by means of the fitting parameters.

Figure 6b,c,e,f show the range shifts produced by the shifted irradiations. The shifts were calculated in terms of the mPAR, sPAR, and PDR with 1σ uncertainty. The 2 and 5 mm range shifter irradiation produced mPAR shifts between 1.4–2.3  and 4.4–5.4 mm for the head plan and 1.6–2.0  and 3.6–4.8 mm for the neck plan; sPAR shifts between 1.3–2.6  and 4.4–4.8 mm for the head plan and 1.1–2.2  and 3.9–4.8 mm for the neck plan. The PDR shifts between 1.0–2.0 mm for the head plan and 3.8–4.8 mm for the neck plan.

When more than one spot is integrated (the second energy layer of the head irradiation and the first and second energy layers of the neck irradiation), the uncertainty of detecting range shifts is reduced to less than 1 mm (Table S9).

## 4 DISCUSSION

As mentioned in Section 1, instantaneous spot‐by‐spot verification based on ^12^N imaging is limited to high‐intensity beam spots early on in the irradiation: high intensity is needed for sufficient ^12^N PET counts and at early times, the ^12^N PET signal is not yet overwhelmed by counts from the longer‐lived ^15^O and ^11^C which have a substantially higher production. In practice, this limits our method to the first energy layer(s), which essentially deliver the dose in the distal edge. In particular cases, a dose distribution could have high‐intensity spots outside of the first few energy layers. Whether ^12^N imaging is useful in such a case will depend on the magnitude of the longer‐lived PET activity accumulated due to the previous energy layers in the region of that spot and will need to be considered on a case‐by‐case basis. The usefulness of ^12^N imaging might, in certain cases, be extended to some extra beam spots by the procedure to obtain pure ^12^N images, which we have used in some of our earlier work.^28^, ^30^

In clinical practice, a patient irradiation typically delivers a target dose of 2 Gy per daily fraction, distributed across 3‐6 fields. Consequently, the most intense field generally delivers no more than 1 Gy to the target. We used 4 Gy irradiations to compensate for the non‐optimal setup in the present work, as explained in Section 2.1.3. The same arguments show how to maximize the number of counts detected per spot, which is crucial to minimizing the PAR uncertainty in a real clinical scenario: maximize the PET scanner sensitivity and optimize the dose delivery. The aspects to optimize include:

1. The shortening of the spot duration in order to avoid the loss of ^12^N counts. For the 4‐Gy irradiations we performed, this represents loss factors of 1.4–2.0. Considering that the PAR uncertainty is inversely proportional to the square root of the number of counts,^30^ the maximum improvement in the PAR uncertainty, in case of a spot duration much shorter than the ^12^N half‐life, would be a factor of ∼1.3.
2. The counting loss due to the saturation of the PET detectors during the beam‐on periods^30^, ^31^ can be avoided by using detectors more tolerant to the high‐count rates generated in these periods. This has been demonstrated to be possible using individual scintillator crystals one‐to‐one coupled to Silicon photomultipliers (SiPM).^28^ Having such high‐count rate detectors relaxes the need for shorter beam spots discussed in point 1. above.
3. Optimizing the PET scanner sensitivity by increasing the solid angle coverage and the detector intrinsic efficiency. The solid angle is increased by setting up more detectors in larger panels. On the other hand, the intrinsic efficiency could be improved by using thicker and/or more efficient scintillation crystals. Some systems that could be promising to meet this objective are the General Electric (GE) digital Omni Legend PET,^42^ which uses 30 mm thick BGO scintillation crystals, resulting in an improvement of 35% on the intrinsic efficiency (i.e., 16% on the PAR uncertainty), and the Human Dynamic Neuro‐Chemical Connectome brain PET scanner using 26 mm thick LSO crystals,^43^ getting an increase of 20% on the intrinsic efficiency (i.e., 10% on the PAR uncertainty).

The MC simulations, essential for the clinical implementation, showed an average deviation between the PAR measurement and simulation of 0.3 mm, that is, about an order of magnitude lower than the experimental PAR uncertainty. This suggests an overestimation of the PAR uncertainty calculated from the uncertainty of the fit parameter. The fit was performed over just a few data points with a fairly large pixel size of 8 mm. Reducing the pixel size to 4 mm so that the fit considers more data points was checked on the simulation images, obtaining a reduction in sPAR uncertainty of a factor of 1.2–1.5. This analysis cannot be performed on the experimental images due to their poor statistics. However, an increased number of counts due to the implementation of points 1. to 3. above might enable smaller pixel sizes, resulting in smaller mPAR uncertainty.

Noteworthy, the ^10^C and ^8^B contributions are not taken into account in the simulations because the data of the cross‐sections that lead to the production of these isotopes are not included in the RayStation MC engine. The ^10^C activity, when it is most intense at the beginning of the irradiation, is of the same order as the ^11^C activity. This rough estimation comes from the fact that the integrated cross‐section ratio of ^10^C‐to‐^11^C is about ∼1/50–60,^44^ while the decay constant (or half‐life) ratio is about ∼60. The activity of ^8^B is not negligible, especially within the energy layer time scale, because its half‐life (770 ms) is similar to the duration of the energy layer switch (700–800 ms). The imaging of this isotope at this time scale could also be used for range monitoring within sub‐second scales but with a larger number of counts detected than in spot‐wise measurements. However, the development of this methodology requires the measurement of the cross‐section of the ^12^C(p,x)^8^B and/or ^16^O(p,x)^8^B reaction channels for their implementation into the MC simulation framework. Finally, the enhanced simulation framework will thus be able to provide predicted ^12^N PET images (with a statistically negligible uncertainty by simulating a sufficient number of protons) in order to perform instantaneous distal edge monitoring during patient irradiation.

Unlike the difference observed between mPAR and sPAR, discussed previously, the difference between mPAR and PDR is mainly systematic. This can be attributed to a combination of physical factors, including the nuclear reaction energy thresholds and the large ^12^N positron range (the 1D projection of the positron range distribution has a root mean square of 1.8 g/cm^2^ in water),^29^ resulting in a distal broadening of the fall‐off region with respect to the positron yield distributions and the PARs of the positron emitters with relatively low positron energies such as ^15^O and ^11^C.

Our results demonstrate the precision of instantaneous in vivo distal edge monitoring by means of spot‐by‐spot PET imaging. It was verified by the ground truth, PDR shifts, which were obtained from the TPS dose distribution. Furthermore, we have validated the PET imaging MC simulation framework for positron activity distal edge prediction.

In a clinical situation, the distal edge verification would only be performed on the spots of the first energy layer(s), so the beam spot delay would be implemented on no more than a few dozen spots. This would mean a maximum increase of a few seconds in the treatment time, which can be managed by the clinic without decreasing patient throughput. The mechanical implementation of the PET scanner in the treatment room is not a bottleneck in the clinical translation of the approach because there are already some implementations in operation that could be applied and adapted to our approach.^26^, ^45^, ^46^

## 5 CONCLUSION

We have experimentally demonstrated the feasibility of performing instantaneous distal edge verification using PET imaging at the level of individual beam spots and within sub‐second time scales. The approach was able to identify treatment deviations, as range shifts, on the same timescales. A target dose of 4 Gy was used such that the experimental setup produces about the same number of counts which an optimized setup would produce during a clinically more realistic 1 Gy irradiation. The individual spot measurements of the PAR showed a precision, at 1.5σ level uncertainty (clinical uncertainty level used in treatment planning), between 1.5 and 3.6 mm, which is equivalent to or less than the robustness margins used in the robust optimization. Additionally, we have shown the validity of a MC simulation framework for spot‐by‐spot PET image prediction, with an average PAR accuracy of 0.4 mm compared to the PAR measurements. We identified several factors to improve the distal edge monitoring performance with an optimized setup, estimating an equivalent PAR uncertainty, but for a multi‐field irradiation of 2 Gy per fraction.

To conclude, this study represents an important step towards the clinical implementation of instantaneous in vivo distal edge verification in IMPT by means of spot‐by‐spot PET imaging. It operates at a sub‐second time scale, making it suitable for use at the beginning of the dose delivery and contributing to the progress of real‐time range verification.

## CONFLICT OF INTEREST STATEMENT

The authors declare no conflicts of interest.

## Supporting information



Supporting Information
