# Supplementary material for: Instantaneous in vivo distal edge verification in intensity‐modulated proton therapy by means of PET imaging
Source: Med Phys. 2025 May 2;52(7):e17850. doi: 10.1002/mp.17850 (PMC12257898; doi:10.1002/mp.17850)
Supplement: Supplementary file 1 — Supporting Information [file MP-52-0-s001.docx]

# SUPPLEMENTARY INFORMATION – FIGURES AND TABLES

| 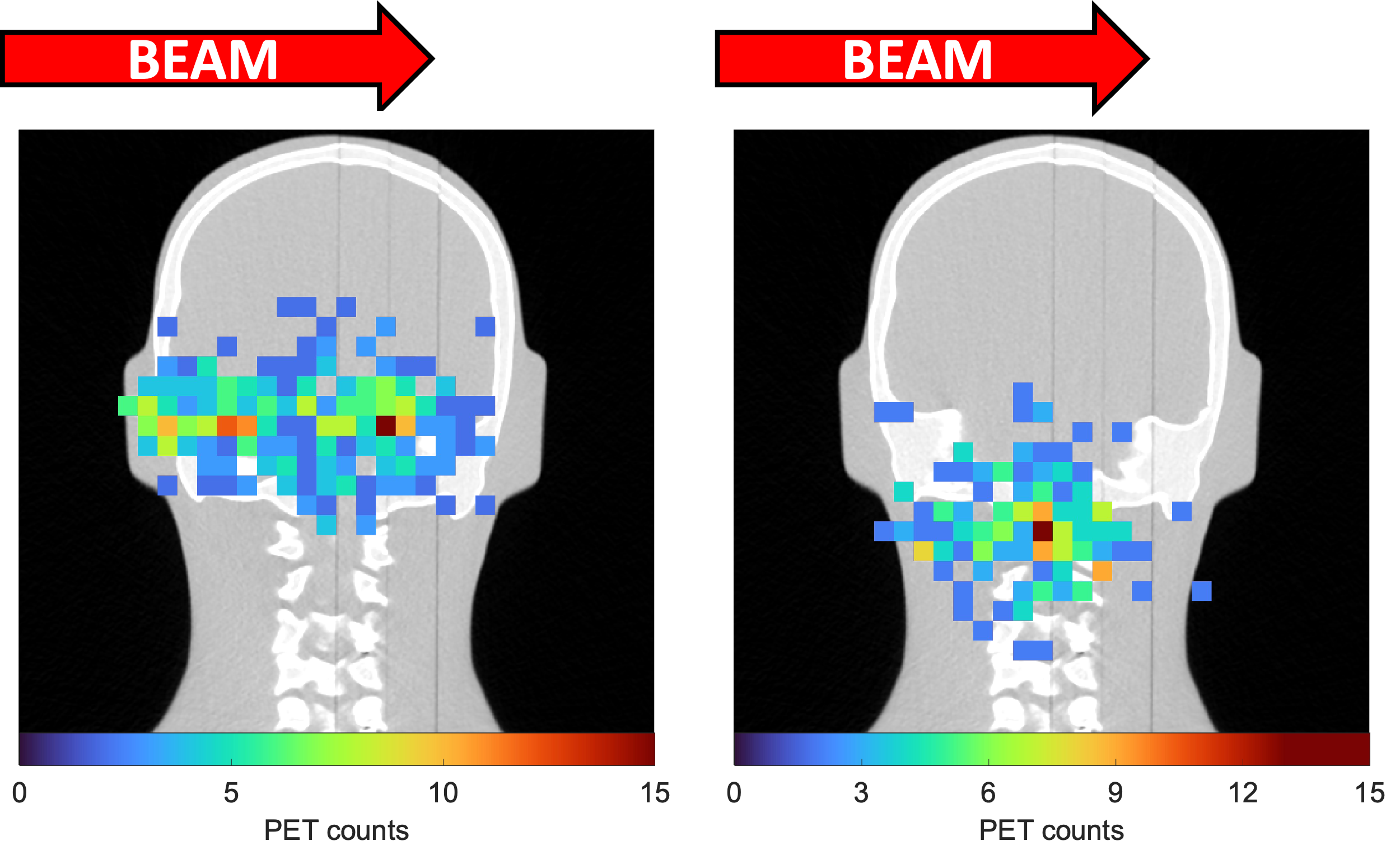  **FIGURE S1**. Individual spot PET image from the first delivered spot in the head (left) with 5.28×10^8^ protons of 146.5 MeV and neck (right) with 2.27×10^8^ protons of 109.3 MeV, overlaid on the phantom CT. |
| --- |

| **TABLE 1.** Relative contributions of the main positron-emitting nuclides to each spot's PET image, obtained from the Monte Carlo simulation. The contributions are shown for the first eight spots, delivered in the first two energy layers, 146.5 and 143.5 MeV, for the head irradiation. The number of protons delivered (and simulated) per spot is also shown. | | | | | |
| --- | --- | --- | --- | --- | --- |
| **Energy layer (MeV)** | **Spot ID** | **No. protons: Plan/Simulated** | **^12^N (%)** | **^11^C (%)** | **^15^O (%)** |
| 146.5 | 01/01 | 5.28×10^8^ / 1.24×10^5^ | 68.8 | 1.4 | 29.8 |
|  |  |  |  |  |  |
| 143.5 | 02/01 | 4.48×10^8^ / 1.05×10^5^ | 66.6 | 1.5 | 31.9 |
|  | 02/02 | 3.91×10^8^ / 0.92×10^5^ | 53.3 | 2.2 | 44.5 |
|  | 02/03 | 4.80×10^8^ / 1.12×10^5^ | 47.4 | 2.4 | 50.2 |
|  | 02/04 | 3.89×10^8^ / 0.91×10^5^ | 37.1 | 3.0 | 59.9 |
|  | 02/05 | 4.59×10^8^ / 0.66×10^5^ | 34.6 | 3.1 | 62.3 |
|  | 02/06 | 2.80×10^8^ / 1.07×10^5^ | 37.5 | 2.9 | 59.6 |
|  | 02/07 | 4.41×10^8^ / 1.03×10^5^ | 27.5 | 3.4 | 69.1 |

| **TABLE 2.** Relative contributions of the main positron-emitting nuclides to each spot's PET image, obtained from the Monte Carlo simulation. The contributions are shown for the first nine spots, delivered in the first two energy layers, 109.3 and 107.0 MeV, for the neck irradiation. The number of protons delivered (and simulated) per spot is also shown. | | | | | |
| --- | --- | --- | --- | --- | --- |
| **Energy layer (MeV)** | **Spot ID** | **No. protons: Plan/Simulated** | **^12^N (%)** | **^11^C (%)** | **^15^O (%)** |
| 109.3 | 01/01 | 2.27×10^8^ / 1.05×10^5^ | 76.2 | 1.2 | 22.7 |
|  | 0/102 | 1.09×10^8^ / 0.50×10^5^ | 72.1 | 1.4 | 26.5 |
|  | 01/03 | 1.34×10^8^ / 0.63×10^5^ | 48.0 | 2.6 | 49.4 |
|  |  |  |  |  |  |
| 107.0 | 02/01 | 3.89×10^8^ / 1.80×10^5^ | 55.2 | 2.3 | 42.6 |
|  | 02/02 | 3.46×10^8^ / 1.60×10^5^ | 37.7 | 3.1 | 59.2 |
|  | 02/03 | 1.69×10^8^ / 0.78×10^5^ | 34.7 | 3.5 | 61.8 |
|  | 02/04 | 1.63×10^8^ / 0.75×10^5^ | 30.7 | 3.7 | 65.6 |
|  | 02/05 | 2.25×10^8^ / 1.04×10^5^ | 29.8 | 3.7 | 66.5 |
|  | 02/06 | 1.84×10^8^ / 0.85×10^5^ | 21.2 | 4.2 | 74.6 |

| **TABLE 3.** mPAR, sPAR and PDR values for the 4-Gy nominal head irradiation. All values are expressed in millimeters, measured from the planning CT isocenter^a^, with their 1σ uncertainty. The fifth column shows the absolute difference between mPARs and sPARs. The last column shows the systematic difference between mPARs and PDRs. | | | | | | | | |
| --- | --- | --- | --- | --- | --- | --- | --- | --- |
| **Energy layer (MeV)** | | **Spot ID** | **mPAR (mm)** | **sPAR (mm)** | **mPAR-sPAR (mm)** | | **PDR (mm)** | **mPAR‑PDR (mm)** |
| 146.5 | | 01/01 | 63.2±1.7 | 62.5±0.8 | 0.7±1.9 | 54.1±0.4 | | 9.1±1.7 |
|  |  | | | | | | | |
| 143.5 | | 02/01 | 59.0±2.2 | 58.9±1.1 | 0.1±2.5 | 52.1±0.3 | | 6.9±2.2 |
|  |  | 02/02 | 59.5±1.3 | 58.9±1.1 | 0.6±1.7 | 52.4±0.3 | | 7.1±1.3 |
|  |  | 02/03 | 55.4±1.2 | 55.0±1.0 | 0.4±1.5 | 48.4±0.3 | | 7.0±1.2 |
|  |  | 02/04 | 58.5±2.0 | 58.2±1.1 | 0.3±2.3 | 50.8±0.3 | | 7.7±2.0 |
|  |  | 02/05 | 52.8±1.1 | 52.7±1.3 | 0.1±1.7 | 45.8±0.2 | | 7.0±1.1 |
|  |  | 02/06 | 57.1±1.4 | 57.0±0.8 | 0.1±1.6 | 50.3±0.3 | | 6.8±1.4 |
|  |  | 02/07 | 56.7±2.0 | 56.4±1.2 | 0.3±2.3 | 49.2±0.4 | | 7.5±2.0 |
| ^a^The z-axis position of the pencil beam entry on the phantom edge was at z=-85.2, -85.1, -84.5, -84.1, -83.4, -79.0, -79.5.0, and -80.0 mm for the 1^st^ to 8^th^ spot, respectively.  Abbreviations: mPAR, measured positron activity range. sPAR, simulated positron activity range. PDR, proton dose range. | | | | | | | | |

| **TABLE 4.** mPAR, sPAR and PDR values for the 4-Gy head irradiation with the 2-mm thickness range shifter. All values are expressed in millimeters, measured from the planning CT isocenter^a^, with their 1σ uncertainty. The fifth column shows the absolute difference between mPARs and sPARs. The last column shows the systematic difference between mPARs and PDRs. | | | | | | | |
| --- | --- | --- | --- | --- | --- | --- | --- |
| **Energy layer (MeV)** | | **Spot ID** | **mPAR (mm)** | **sPAR (mm)** | **mPAR-sPAR (mm)** | **PDR (mm)** | **mPAR‑PDR (mm)** |
| 146.5 | | 01/01 | 61.8±2.4 | 61.2±1.0 | 0.6±2.6 | 52.3±0.3 | 9.5±2.4 |
|  |  | | | | | | |
| 143.5 | | 02/01 | 57.1±2.3 | 56.9±1.1 | 0.2±2.5 | 50.1±0.3 | 7.0±2.3 |
|  |  | 02/02 | 57.2±1.7 | 56.5±1.2 | 0.7±2.1 | 50.4±0.3 | 6.8±1.7 |
|  |  | 02/03 | 53.5±1.6 | 53.0±1.2 | 0.5±2.0 | 46.9±0.3 | 6.6±1.6 |
|  |  | 02/04 | 56.4±2.3 | 56.4±0.8 | 0.0±2.4 | 49.3±0.3 | 7.1±2.3 |
|  |  | 02/05 | 50.7±1.6 | 51.0±1.0 | -0.3±1.9 | 44.3±0.4 | 6.4±1.6 |
|  |  | 02/06 | 55.1±2.0 | 54.4±1.2 | 0.7±2.3 | 48.5±0.3 | 6.6±2.0 |
|  |  | 02/07 | 54.4±2.1 | 54.8±0.8 | -0.4±2.3 | 47.6±0.3 | 6.8±2.1 |
| ^a^The z-axis position of the pencil beam entry on the phantom edge was at z=-85.2, -85.1, -84.5, -84.1, -83.4, -79.0, -79.5.0, and -80.0 mm for the 1^st^ to 8^th^ spot, respectively.  Abbreviations: mPAR, measured positron activity range. sPAR, simulated positron activity range. PDR, proton dose range. | | | | | | | |

| **TABLE 5.** mPAR, sPAR and PDR values for the 4-Gy head irradiation with the 5-mm thickness range shifter. All values are expressed in millimeters, measured from the planning CT isocenter^a^, with their 1σ uncertainty. The fifth column shows the absolute difference between mPARs and sPARs. The last column shows the systematic difference between mPARs and PDRs. | | | | | | | |
| --- | --- | --- | --- | --- | --- | --- | --- |
| **Energy layer (MeV)** | | **Spot ID** | **mPAR (mm)** | **sPAR (mm)** | **mPAR-sPAR (mm)** | **PDR (mm)** | **mPAR‑PDR (mm)** |
| 146.5 | | 01/01 | 58.8±2.4 | 57.8±0.8 | 1.0±2.5 | 49.8±0.2 | 9.0±2.4 |
|  |  | | | | | | |
| 143.5 | | 02/01 | 54.4±2.6 | 54.2±0.9 | 0.2±2.7 | 47.3±0.2 | 7.1±2.6 |
|  |  | 02/02 | 54.8±2.2 | 54.1±1.0 | 0.7±2.4 | 47.6±0.3 | 7.2±2.2 |
|  |  | 02/03 | 50.6±2.5 | 50.2±0.8 | 0.4±2.6 | 43.7±0.4 | 6.9±2.5 |
|  |  | 02/04 | 54.0±1.1 | 53.6±1.1 | 0.4±1.5 | 46.8±0.2 | 7.2±1.1 |
|  |  | 02/05 | 48.1±1.9 | 48.3±0.7 | -0.2±2.0 | 41.9±0.3 | 6.2±1.9 |
|  |  | 02/06 | 52.6±1.4 | 52.5±0.8 | 0.1±1.6 | 46.0±0.3 | 6.6±1.4 |
|  |  | 02/07 | 51.3±1.1 | 51.9±1.1 | -0.6±1.6 | 45.5±0.4 | 5.8±1.1 |
| ^a^The z-axis position of the pencil beam entry on the phantom edge was at z=-85.2, -85.1, -84.5, -84.1, -83.4, -79.0, -79.5.0, and -80.0 mm for the 1^st^ to 8^th^ spot, respectively.  Abbreviations: mPAR, measured positron activity range. sPAR, simulated positron activity range. PDR, proton dose range. | | | | | | | |

| **TABLE 6.** mPAR, sPAR and PDR values for the 4-Gy nominal neck irradiation. All values are expressed in millimeters, measured from the planning CT isocenter^a^, with their 1σ uncertainty. The fifth column shows the absolute difference between mPARs and sPARs. The last column shows the systematic difference between mPARs and PDRs. | | | | | | | |
| --- | --- | --- | --- | --- | --- | --- | --- |
| **Energy layer (MeV)** | | **Spot ID** | **mPAR (mm)** | **sPAR (mm)** | **mPAR-sPAR (mm)** | **PDR (mm)** | **mPAR‑PDR (mm)** |
| 109.3 | | 01/01 | 27.3±1.8 | 28.0±0.6 | -0.7±1.9 | 20.5±0.3 | 6.8±1.8 |
|  |  | 0/102 | 24.6±1.4 | 24.4±0.4 | 0.2±1.4 | 18.0±0.3 | 6.6±1.4 |
|  |  | 01/03 | 24.4±1.0 | 24.5±0.8 | -0.1±1.3 | 18.5±0.4 | 5.9±1.1 |
|  |  | | | | | | |
| 107.0 | | 02/01 | 23.4±1.1 | 23.1±1.1 | 0.3±1.5 | 16.6±0.2 | 6.8±1.1 |
|  |  | 02/02 | 21.5±1.2 | 20.8±0.9 | 0.7±1.5 | 14.0±0.4 | 7.5±1.3 |
|  |  | 02/03 | 21.9±1.9 | 22.0±0.9 | -0.1±2.1 | 15.7±0.4 | 6.2±1.9 |
|  |  | 02/04 | 21.0±1.7 | 20.6±1.0 | 0.4±2.0 | 14.9±0.3 | 6.1±1.7 |
|  |  | 02/05 | 21.3±1.3 | 21.2±1.1 | 0.1±1.7 | 15.3±0.3 | 6.0±1.3 |
|  |  | 02/06 | 21.4±1.0 | 21.6±1.3 | -0.2±1.7 | 15.3±0.4 | 6.1±1.0 |
| ^a^The z-axis position of the pencil beam entry on the phantom edge was at z=-60.9, -62.6, -64.1, -58.7, -60.9, -62.3, -64.4, ‑62.6, and -64.2 mm for the 1^st^ to 9^th^ spot, respectively.  Abbreviations: mPAR, measured positron activity range. sPAR, simulated positron activity range. PDR, proton dose range. | | | | | | | |

| **TABLE 7.** mPAR, sPAR and PDR values for the 4-Gy neck irradiation with the 2-mm thickness range shifter. All values are expressed in millimeters, measured from the planning CT isocenter^a^, with their 1σ uncertainty. The fifth column shows the absolute difference between mPARs and sPARs. The last column shows the systematic difference between mPARs and PDRs. | | | | | | | |
| --- | --- | --- | --- | --- | --- | --- | --- |
| **Energy layer (MeV)** | | **Spot ID** | **mPAR (mm)** | **sPAR (mm)** | **mPAR-sPAR (mm)** | **PDR (mm)** | **mPAR‑PDR (mm)** |
| 146.5 | | 01/01 | 25.7±1.6 | 26.5±0.6 | -0.8±1.7 | 19.5±0.2 | 6.2±1.6 |
|  |  | 0/102 | 22.8±1.1 | 22.8±0.6 | 0.0±1.2 | 16.5±0.29 | 6.3±1.1 |
|  |  | 01/03 | 22.8±1.0 | 22.7±0.7 | 0.1±1.2 | 16.5±0.24 | 6.3±1.0 |
|  |  | | | | | | |
| 143.5 | | 02/01 | 21.7±1.3 | 21.9±1.2 | -0.2±1.8 | 15.0±0.39 | 6.7±1.4 |
|  |  | 02/02 | 19.8±14 | 19.4±1.2 | 0.4±1.8 | 12.5±0.24 | 7.3±1.4 |
|  |  | 02/03 | 20.3±1.9 | 19.8±1.1 | 0.5±2.2 | 14.0±0.27 | 6.3±1.9 |
|  |  | 02/04 | 19.0±1.4 | 18.8±1.2 | 0.2±1.8 | 13.1±0.25 | 5.9±1.4 |
|  |  | 02/05 | 20.3±1.3 | 20.1±1.4 | 0.2±1.9 | 13.7±0.38 | 6.6±1.4 |
|  |  | 02/06 | 20.0±1.3 | 19.8±1.4 | 0.2±1.9 | 13.4±0.31 | 6.6±1.3 |
| ^a^The z-axis position of the pencil beam entry on the phantom edge was at z=-60.9, -62.6, -64.1, -58.7, -60.9, -62.3, -64.4, ‑62.6, and -64.2 mm for the 1^st^ to 9^th^ spot, respectively.  Abbreviations: mPAR, measured positron activity range. sPAR, simulated positron activity range. PDR, proton dose range. | | | | | | | |

| **TABLE 8.** mPAR, sPAR and PDR values for the 4-Gy neck irradiation with the 5-mm thickness range shifter. All values are expressed in millimeters, measured from the planning CT isocenter^a^, with their 1σ uncertainty. The fifth column shows the absolute difference between mPARs and sPARs. The last column shows the systematic difference between mPARs and PDRs. | | | | | | | |
| --- | --- | --- | --- | --- | --- | --- | --- |
| **Energy layer (MeV)** | | **Spot ID** | **mPAR (mm)** | **sPAR (mm)** | **mPAR-sPAR (mm)** | **PDR (mm)** | **mPAR‑PDR (mm)** |
| 109.3 | | 01/01 | 23.1±1.1 | 24.1±0.7 | -1.0±1.3 | 16.6±0.4 | 6.5±1.2 |
|  |  | 01/02 | 20.2±1.6 | 20.2±0.6 | 0.0±1.7 | 14.1±0.3 | 6.1±1.6 |
|  |  | 01/03 | 19.6±1.4 | 19.7±0.7 | -0.1±1.6 | 13.1±0.3 | 6.5±1.4 |
|  |  | | | | | | |
| 107.0 | | 02/01 | 19.4±1.3 | 19.2±1.3 | 0.2±1.8 | 12.8±0.2 | 6.6±1.3 |
|  |  | 02/02 | 17.1±1.5 | 16.4±1.0 | 0.7±1.8 | 10.0±0.3 | 7.1±1.5 |
|  |  | 02/03 | 17.7±1.5 | 18.0±1.1 | -0.3±1.9 | 11.6±0.4 | 6.1±1.5 |
|  |  | 02/04 | 16.5±1.7 | 16.2±1.0 | 0.3±2.0 | 10.7±0.3 | 5.8±1.7 |
|  |  | 02/05 | 17.7±1.5 | 17.3±1.4 | 0.4±2.1 | 11.1±0.4 | 6.6±1.5 |
|  |  | 02/06 | 17.0±1.8 | 16.9±1.4 | 0.1±2.3 | 10.5±0.2 | 6.5±1.8 |
| ^a^The z-axis position of the pencil beam entry on the phantom edge was at z=-60.9, -62.6, -64.1, -58.7, -60.9, -62.3, -64.4, ‑62.6, and -64.2 mm for the 1^st^ to 9^th^ spot, respectively.  Abbreviations: mPAR, measured positron activity range. sPAR, simulated positron activity range. PDR, proton dose range. | | | | | | | |

| **TABLE 9**. Positron activity and proton range shifts, and their 1σ uncertainty, averaged over all spots of the same energy layer for the first two energy layers using the 2-mm and 5-mm range shifters. | | | | | |
| --- | --- | --- | --- | --- | --- |
| **Energy layer (MeV)** | **No. protons (×10^8^)** | **Range shifter thickness** | **ΔmPAR (mm)** | **ΔsPAR (mm)** | **ΔPDR (mm)** |
| 146.5 (head) | 5.28 | 2-mm | 1.6±2.9 | 1.3±1.3 | 1.8±0.5 |
|  |  | 5-mm | 4.6±2.9 | 4.7±1.1 | 4.3±0.4 |
|  | | | | | |
| 143.5 (head) | 28.9 | 2-mm | 2.1±1.0 | 1.9±0.6 | 1.7±0.2 |
|  |  | 5-mm | 4.7±1.0 | 4.6±0.5 | 4.3±0.2 |
|  | | | | | |
| 109.3 (neck) | 4.7 | 2-mm | 1.7±1.1 | 1.6±0.5 | 1.3±0.2 |
|  |  | 5-mm | 4.5±1.2 | 4.3±0.5 | 3.9±0.2 |
|  | | | | | |
| 107.0 (neck) | 14.8 | 2-mm | 1.6±0.9 | 1.6±0.7 | 1.7±0.2 |
|  |  | 5-mm | 4.2±0.9 | 4.2±0.6 | 4.3±0.2 |
| Abbreviations: mPAR, measured positron activity range. sPAR, simulated positron activity range. PDR, proton dose range. ΔmPAR, measured positron activity range shift. ΔsPAR, simulated positron activity range shift. ΔmPDR, proton dose range shift. | | | | | |
